# A Comprehensive Review of Prone Ventilation in the Intensive Care Unit: Challenges and Solutions

**DOI:** 10.7759/cureus.57247

**Published:** 2024-03-30

**Authors:** Vishnu Priya, Jayashree Sen, Sanjot Ninave

**Affiliations:** 1 Anesthesiology, Jawaharlal Nehru Medical College, Datta Meghe Institute of Higher Education & Research, Wardha, IND

**Keywords:** patient-centered care, technological innovations, challenges, physiological rationale, intensive care unit (icu), prone ventilation

## Abstract

This comprehensive review explores the intricate landscape of prone ventilation in the intensive care unit (ICU), spanning physiological rationale, challenges in implementation, psychosocial impacts, technological innovations, economic considerations, barriers to adoption, and implications for clinical practice. The physiological benefits of prone positioning, including improved oxygenation and lung compliance, are discussed alongside the challenges of patient selection and technical complexities. The psychosocial impact on patients and caregivers, as well as the economic implications for healthcare systems, adds a crucial dimension to the analysis. The review also delves into innovative technologies, such as advanced monitoring and automation, shaping the landscape of prone ventilation. Moreover, it addresses the barriers to widespread adoption and outlines strategies to overcome resistance, emphasizing the need for a comprehensive and collaborative approach. The implications for clinical practice underscore the importance of evidence-based guidelines, ongoing education, and a holistic patient-centered care approach. The conclusion highlights the call to action for further research to refine protocols and technology, ultimately optimizing the application of prone ventilation in critical care settings.

## Introduction and background

Mechanical ventilation serves as a cornerstone in the management of critically ill patients within the intensive care unit (ICU). This life-saving intervention plays a pivotal role in supporting patients with respiratory failure, providing them with the necessary oxygenation and ventilation when their natural respiratory efforts prove insufficient. However, despite its crucial role, conventional mechanical ventilation is not without limitations, particularly in cases where patients exhibit severe hypoxemia or struggle with maintaining optimal lung compliance [1].

Prone ventilation emerges as a compelling strategy to address the challenges posed by conventional supine mechanical ventilation. Unlike the conventional approach where patients lie on their backs, prone ventilation involves positioning patients face-down. This seemingly simple adjustment in body position has demonstrated remarkable effects on oxygenation, ventilation-perfusion matching, and overall lung mechanics [2].

The adoption of prone ventilation is particularly notable in specific patient populations characterized by severe respiratory distress, such as those suffering from acute respiratory distress syndrome (ARDS). The anatomical and physiological benefits of prone positioning have been observed to enhance oxygenation and potentially improve clinical outcomes in these critically ill individuals. Understanding the contexts in which prone positioning proves most beneficial is integral to optimizing its application in the ICU [3].

The purpose of this comprehensive review is to delve into the intricacies of prone ventilation in the ICU, exploring its physiological underpinnings, clinical efficacy, and the challenges encountered in its implementation. By critically examining existing literature, we aim to identify the key advantages and limitations of prone ventilation, shedding light on the specific patient populations that stand to benefit the most. Moreover, this review aims to provide insights into the evolving landscape of prone ventilation, encompassing technological innovations, educational imperatives, and potential avenues for future research. Through this exploration, we seek to equip healthcare professionals and researchers with a nuanced understanding of prone ventilation's role in intensive care, paving the way for informed decision-making and improved patient outcomes.

## Review

Physiological rationale for prone ventilation

Mechanisms of Improved Oxygenation

**Figure 1:**
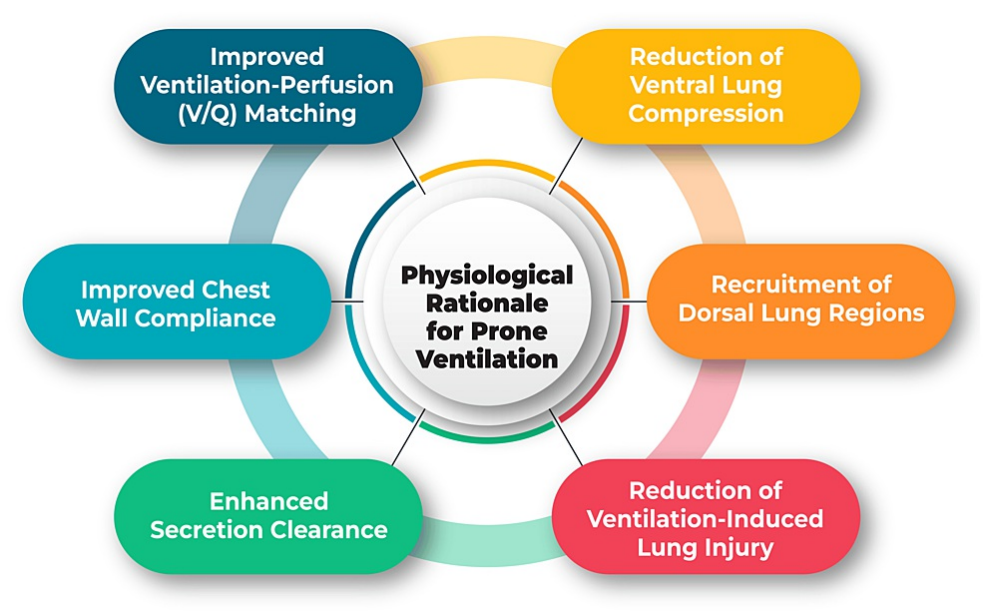
Physiological rationale for prone ventilation The author has self-created image.

The enhancement of oxygenation is a central physiological benefit associated with prone ventilation. In the prone position, gravitational forces influence lung perfusion differently, leading to more uniform ventilation distribution. This redistribution of blood flow and a reduction in lung compression contribute to a more homogenous ventilation-perfusion ratio across the lungs. Additionally, prone positioning has been shown to mitigate ventilation-perfusion mismatching, a common challenge in patients with severe respiratory compromise [4]. Ventilation in the dependent lung regions, which often experience better perfusion in the prone position, improves, thereby optimizing gas exchange. Alveoli in the dorsal lung regions tend to be more recruited and less prone to collapse, fostering improved oxygen diffusion and delivery to the bloodstream. The net effect is an amelioration of oxygenation parameters, a critical aspect in managing patients with conditions characterized by refractory hypoxemia [5]. The physiological rationale for prone ventilation is shown in Figure 1.

Impact on Ventilation-Perfusion Matching

Prone ventilation profoundly influences the matching of ventilation and perfusion, addressing one of the key challenges in respiratory physiology. In the supine position, gravity induces uneven distribution of blood flow and ventilation, leading to areas of the lung that are over- or under-ventilated relative to their perfusion. Prone positioning counteracts these gravitational effects, promoting more uniform ventilation and perfusion across lung regions [6]. This improved matching is particularly relevant in conditions where lung injury disrupts the normal matching process, such as ARDS. By aligning ventilation and perfusion more closely, prone ventilation helps optimize gas exchange efficiency, potentially alleviating hypoxemia and reducing reliance on higher positive end-expiratory pressure (PEEP) [7].

Effects on Lung Compliance and Recruitment

The prone position positively influences lung compliance and recruitment, which are critical factors in managing patients with compromised respiratory function. In the supine position, the weight of the heart's and mediastinal structures can impede lung expansion, leading to atelectasis and decreased lung compliance. Prone positioning mitigates this effect by allowing the heart and mediastinum to rest against the sternum, freeing up the dorsal lung regions for improved expansion [8]. Furthermore, the prone position facilitates a more homogeneous distribution of stress and strain across the lung parenchyma. This can lead to the recruitment of collapsed or atelectatic alveoli, improving overall lung compliance and enhancing the potential for lung-protective ventilation strategies [4]. Understanding these physiological mechanisms is paramount for clinicians implementing prone ventilation in the ICU, as it forms the foundation for its clinical efficacy and informs the rationale behind its application in specific patient populations [9].

Clinical evidence and outcomes

Review of Clinical Trials and Studies

Prone positioning in adults with ARDS through manual intervention can elevate the likelihood of complications, such as pressure injuries, significant airway issues, ocular and nerve injuries, and complications related to enteral feeding [10]. It is crucial to implement evidence-based strategies to prevent these complications and ensure secure handling for the well-being of caregivers. Prone ventilation has been linked to a reduction in mortality among ARDS patients. Research indicates that employing prone positioning for at least 16 hours daily can significantly decrease 90-day mortality without substantial adverse effects [3]. This underscores the potential life-saving impact of prone ventilation in critical care settings. When combined with low tidal volume ventilation, prone positioning may exhibit synergistic lung-protective effects. The survival advantage of the prone position seems contingent on the concurrent use of low tidal volumes [3]. Extended prone position ventilation has been deemed feasible and relatively safe for treating critically ill patients with ARDS, including those with COVID-19-related ARDS. This suggests potential implications for a broader acceptance of prone ventilation in ARDS management [11]. A randomized clinical trial focusing on awake prone positioning in COVID-19 patients with acute respiratory failure discovered that prone positioning did not significantly impact the number of days free from invasive mechanical or noninvasive ventilation at 30 days. Nevertheless, there were no reports of serious adverse events in either group, indicating the safety of this intervention [12].

Challenges in implementing prone ventilation

Patient Selection Criteria

Effective implementation of prone ventilation necessitates careful consideration of patient selection criteria. Not all patients with respiratory failure may benefit equally from prone positioning; identifying those who stand to gain the most is critical. Patient-specific factors must be considered, such as the severity of hypoxemia, the underlying etiology of respiratory failure, and hemodynamic stability. Striking a balance between the potential benefits and the risks associated with prone positioning requires a nuanced understanding of the patient's clinical status [13]. The challenge lies in developing standardized, evidence-based protocols for patient selection that can be readily applied in diverse clinical settings. Additionally, ongoing assessment and reassessment during prone ventilation are essential to ensure its continued appropriateness for the patient's evolving condition [14-18].

Technical Challenges in the Prone Positioning Process

The physical act of transitioning a patient into a prone position introduces a set of technical challenges. Prone positioning involves a coordinated effort by a multidisciplinary team and requires specialized training. Logistical considerations, such as the availability of trained personnel, appropriate equipment, and dedicated space within the ICU, play a crucial role in successfully executing prone ventilation [18]. Ensuring patient safety during the turning process is paramount, as it involves the potential for dislodgement of life-sustaining equipment, hemodynamic instability, and pressure injuries. Addressing these technical challenges involves proper training and the integration of ergonomic aids and equipment designed to facilitate a smooth and secure transition into the prone position [18-20].

Staffing and Resource Considerations

Implementing prone ventilation places significant demands on staffing and resources within the ICU. A successful prone positioning strategy requires a well-coordinated team of healthcare professionals, including respiratory therapists, nurses, and physicians. The need for additional personnel during the turning procedure and ongoing monitoring necessitates careful consideration of staffing levels to ensure patient safety and the efficient use of resources [21]. Furthermore, resource considerations extend beyond personnel to include the availability of specialized equipment, such as prone beds or cushions, and the overall infrastructure of the ICU. Adequate training programs and ongoing education are crucial to address staffing challenges and ensure a standardized approach to prone ventilation [22].

Monitoring and Managing Complications

Prone ventilation introduces unique challenges related to monitoring and managing complications. While the prone position can improve oxygenation and lung mechanics, it has potential risks. Complications such as pressure sores, endotracheal tube displacement, and cardiovascular instability may arise and require vigilant monitoring [23]. Implementing robust monitoring protocols is essential to identify and address complications promptly. This includes continuous assessment of hemodynamic parameters, maintaining secure airway management, and proactive measures to prevent pressure-related injuries. A comprehensive understanding of potential complications and establishing standardized protocols for their prevention and management are imperative to ensure the safety and efficacy of prone ventilation in the ICU [24].

Psychosocial impact on patients and caregivers

Patient Experience During Prone Ventilation

The experience of prone ventilation goes beyond the physiological realm and significantly influences patients' psychosocial well-being. Being positioned face-down for an extended period can be disorienting and emotionally challenging. Patients may experience anxiety, a sense of isolation, or feelings of vulnerability. Understanding and addressing the psychological impact of prone ventilation on patients is integral to providing holistic and patient-centered care [2]. Enhancing the patient experience should encompass effective communication, providing clear information about the rationale behind prone positioning, and offering psychological support. Incorporating interventions such as music, relaxation techniques, or virtual reality may help mitigate the stress associated with the prone position [25].

Impact on Caregiver and Family Interactions

The psychosocial impact of prone ventilation extends to caregivers and family members who play a crucial role in the support network of critically ill patients. The physical repositioning of the patient can alter the dynamics of caregiver interactions, making communication and emotional support more challenging. Caregivers may experience increased stress and uncertainty about the well-being of their loved ones [26]. Efforts to mitigate the impact on caregivers involve transparent communication regarding the goals and potential challenges of prone ventilation. Providing resources for emotional support, such as counseling services or support groups, can help caregivers navigate the unique challenges associated with caring for a loved one undergoing prone ventilation [27].

Addressing Psychological Challenges for Both Patients and Caregivers

Recognizing and addressing the psychological challenges faced by both patients and caregivers during prone ventilation is essential for comprehensive care. Implementing psychosocial support programs within the ICU setting can include regular communication with patients about their experiences, addressing fears and anxieties, and involving mental health professionals when needed [28]. For caregivers, establishing a supportive environment that encourages open communication with the healthcare team is crucial. Educational initiatives that explain the rationale behind prone ventilation and prepare caregivers for potential emotional challenges can contribute to a more positive experience [27]. Furthermore, integrating family-centered care models, where caregivers are actively involved in the care process, can foster a sense of inclusion and empower them to cope with the unique demands of prone ventilation [27].

Innovations and technological advances

Advances in Prone Positioning Devices

Recent years have witnessed notable advancements in developing prone positioning devices to enhance the safety and comfort of patients undergoing prone ventilation. Traditional manual methods of turning patients into prone positions can be labor-intensive and pose risks, particularly in critically ill individuals. Innovations in prone positioning devices include specialized beds and cushions to facilitate a controlled and ergonomic transition [29]. These devices are engineered to provide optimal support, distribute pressure evenly, and minimize the risk of complications such as pressure ulcers. Some incorporate adjustable elements, allowing healthcare providers to customize the degree of pronation to meet individual patient needs. Evaluating the efficacy and safety of these devices in various clinical settings is crucial for their widespread adoption and integration into routine ICU practices [30].

Monitoring Technologies for Prone Ventilation

Monitoring patients during prone ventilation is essential for ensuring safety and optimizing therapeutic outcomes. Technological advances in monitoring devices have played a pivotal role in enhancing the precision and efficiency of patient management in the prone position. Continuous monitoring of vital signs, such as heart rate, blood pressure, and oxygen saturation, has become more sophisticated, providing real-time data to guide clinical decision-making [31]. Furthermore, imaging technologies, such as electrical impedance tomography and lung ultrasound, have shown promise in assessing lung aeration and ventilation distribution during prone ventilation. These tools offer valuable insights into the physiological effects of prone positioning, allowing for prompt adjustments to ventilation strategies [32]. Integrating electronic health record systems with monitoring technologies also streamlines data analysis and facilitates a more comprehensive understanding of patient responses to prone ventilation. The ongoing refinement and validation of these monitoring tools are crucial for their effective implementation in diverse clinical scenarios [32].

Automation and Robotics in Prone Positioning

Integrating automation and robotics represents a frontier of innovation in prone positioning techniques. Robotic-assisted systems offer the potential to standardize and streamline the process of turning patients into prone positions, reducing the physical burden on healthcare providers and enhancing the precision of the maneuver [33]. These systems can be programmed to execute controlled and reproducible prone positioning, ensuring patients' consistent and safe transition. Additionally, robotics may play a role in maintaining patient stability during the turning process, minimizing the risk of complications. While in the early stages of development and adoption, the potential benefits of automation and robotics in prone positioning underscore the need for further research and evaluation of their efficacy and safety in diverse clinical contexts [34].

Training and education for healthcare professionals

Importance of Education in Prone Ventilation

Education is a cornerstone in successfully implementing prone ventilation in intensive care. Healthcare professionals involved in critically ill patients' care must comprehensively understand the physiological principles, indications, and potential complications associated with prone positioning. Adequate education lays the foundation for safe and effective application, fostering confidence and competence among the healthcare team [35]. Structured educational programs should cover theoretical and practical aspects, including patient selection criteria, turning techniques, and complication management. Recognizing the dynamic nature of critical care, ongoing education ensures that healthcare professionals remain abreast of the latest advancements and evidence in prone ventilation [36].

Simulation Training for Healthcare Professionals

Simulation training is a valuable tool in preparing healthcare professionals for the challenges associated with prone ventilation. Simulated scenarios offer a controlled environment where practitioners can practice and refine their skills risk-free. This includes the coordination required for turning patients, troubleshooting potential complications, and optimizing patient care during prone ventilation [37]. Simulation training enhances technical proficiency and promotes effective communication and teamwork among healthcare team members. It allows for rehearsing protocols and developing standardized procedures, contributing to a more cohesive and prepared response in real-life clinical situations [38]. Additionally, integrating virtual and augmented reality technologies can provide immersive training experiences, allowing healthcare professionals to navigate complex scenarios and build confidence in managing prone ventilation effectively [38].

Continuous Learning and Updates on Best Practices

The landscape of critical care is dynamic, with continuous advancements in medical knowledge and technology. To ensure the consistent delivery of high-quality care in prone ventilation, healthcare professionals must engage in ongoing learning and stay updated on best practices [39]. Regular training sessions, workshops, and educational conferences on prone ventilation should be part of professional development initiatives. These forums facilitate the exchange of knowledge, experiences, and evidence-based practices among healthcare professionals. Moreover, they provide a platform for discussing emerging research, technological innovations, and updates to clinical guidelines related to prone ventilation [39]. Establishing mentorship programs and peer-learning opportunities further contributes to the dissemination of expertise and the cultivation of a culture of continuous improvement within the healthcare community [39].

Economic considerations

Cost-Effectiveness of Prone Ventilation

Understanding the cost-effectiveness of prone ventilation is essential for healthcare decision-makers and administrators. While prone ventilation has demonstrated clinical benefits, it is imperative to evaluate its economic implications. This involves assessing the costs associated with specialized equipment, personnel training, and potential complications against the savings or benefits derived from improved patient outcomes [40]. Economic analyses should consider factors such as the reduction in ventilator days, decreased incidence of complications, and overall impact on the patient's length of stay in the ICU. Evaluating the cost-effectiveness of prone ventilation contributes to informed resource allocation and aids in decision-making processes related to the adoption and sustainability of this intervention [40].

Impact on Length of Stay and Resource Utilization

Prone ventilation can potentially influence the ICU's length of stay and resource utilization. If prone positioning proves to expedite recovery and decrease the duration of mechanical ventilation, it may lead to shorter ICU stays and reduced resource consumption. Conversely, challenges such as the need for specialized training and additional monitoring may contribute to increased resource utilization [29]. Assessing the impact on the length of stay involves direct costs related to ICU resources and indirect costs associated with potential complications and extended hospitalization. Understanding these dynamics is crucial for healthcare institutions aiming to optimize resource allocation and manage patient flow efficiently [29].

Economic Implications for Healthcare Systems

On a broader scale, the economic implications of prone ventilation extend to healthcare systems. Hospitals and healthcare providers must consider the overall financial impact, such as reimbursement models, budget constraints, and the potential return on investment [39]. Evaluating the economic viability of prone ventilation requires a comprehensive analysis of the associated costs and benefits. This includes direct costs related to equipment, training, and personnel, as well as indirect costs tied to changes in patient outcomes, readmission rates, and long-term healthcare utilization [39]. Health economic studies and analyses provide decision-makers with the necessary data to determine the economic feasibility of incorporating prone ventilation into standard ICU practices. This information informs strategic planning, budgeting, and policy development within healthcare systems, ensuring financial resources are allocated judiciously to interventions that yield the greatest value [39].

Barriers to adoption

Identifying and Addressing Barriers to Widespread Use

Despite the proven clinical benefits of prone ventilation, several barriers hinder its widespread adoption in the intensive care setting. Identifying these barriers is crucial for devising effective strategies to overcome them. Common obstacles include a lack of standardized protocols, insufficient training, and concerns regarding potential complications [16]. Addressing these barriers requires a multifaceted approach. Establishing evidence-based guidelines and protocols for prone ventilation can provide a foundation for consistent practice. Comprehensive training programs that address the technical and logistical aspects of prone positioning are essential. Additionally, fostering a culture of open communication and collaboration among healthcare professionals can facilitate the sharing of best practices and insights, breaking down barriers to adoption [41].

Cultural and Institutional Challenges

Cultural and institutional factors play a significant role in accepting and integrating prone ventilation into routine clinical practice. Resistance to change, established norms, and ingrained practices can create cultural barriers within healthcare institutions. Institutional challenges may include limited resources, stakeholder resistance, and a lack of buy-in from key decision-makers [42]. Strategies to overcome cultural and institutional challenges involve cultivating a culture of innovation and continuous improvement. Leadership support is crucial in championing the adoption of prone ventilation, emphasizing its potential impact on patient outcomes and the overall quality of care. Collaborative decision-making involving all relevant stakeholders can help address concerns and build consensus [43].

Strategies for Overcoming Resistance to Change

Education and training: Comprehensive education and training programs form the foundation for successfully implementing prone ventilation in healthcare settings. These programs should encompass a thorough understanding of the physiological principles, practical aspects, and potential complications associated with prone positioning. Hands-on training sessions and workshops allow healthcare professionals to develop and refine the skills required for safe and effective ventilation. Ongoing educational initiatives are essential to keep healthcare professionals updated on the latest evidence, best practices, and technological advancements [2].

Engaging key stakeholders: The active involvement of key stakeholders, including physicians, nurses, respiratory therapists, and administrators, is crucial for overcoming resistance to change and fostering a collaborative approach to prone ventilation. Open and transparent dialogue is essential to address concerns, share insights, and build consensus regarding integrating prone ventilation into clinical practice. By involving stakeholders in decision-making, healthcare institutions can create a sense of ownership and shared responsibility, ultimately facilitating a smoother transition [42].

Pilot programs and implementation teams: Introducing prone ventilation through pilot programs provides a controlled and phased approach to testing and adjusting protocols before full-scale implementation. Dedicated implementation teams are pivotal in providing ongoing support to frontline staff, troubleshooting challenges as they arise, and gathering valuable feedback. This iterative process identifies and resolves potential issues, ensuring a more seamless and effective integration of prone ventilation into routine clinical care [42].

Performance feedback and quality improvement: Implementing mechanisms for continuous performance feedback and quality improvement is essential for refining prone ventilation practices over time. Regular evaluation of outcomes, complication rates, and protocol adherence provide valuable insights for ongoing optimization. This data-driven approach enables healthcare institutions to identify areas for improvement, implement targeted interventions, and consistently deliver high-quality care [42].

Patient and family education: Involving patients and their families in the decision-making process and providing education about the benefits of prone ventilation is crucial for building a supportive environment. Informed and engaged patients are more likely to understand the rationale behind prone positioning, actively participate in their care, and contribute to a positive healthcare experience. Patient and family education programs should emphasize clear communication, address concerns, and empower individuals to partner in their healthcare journey [43].

## Conclusions

In conclusion, this thorough review has delved into the complexities surrounding prone ventilation within the ICU. Unveiling its physiological underpinnings, the discussion highlighted the potential of prone positioning to ameliorate oxygenation, ventilation-perfusion disparities, and lung compliance. However, the implementation of this technique presents a spectrum of challenges, ranging from patient selection nuances to the intricacies of the turning process. The psychosocial impact on patients and caregivers cannot be understated, emphasizing the importance of a holistic approach to critical care. Moreover, technological innovations, economic considerations, and institutional barriers underscore the multifaceted nature of adopting prone ventilation. The implications for clinical practice call for a nuanced and evidence-based approach, integrating ongoing education and simulation training. As we conclude, a resounding call to action resonates-further research is imperative to refine protocols, enhance technology, and address long-term outcomes. By embracing a comprehensive and collaborative strategy, healthcare professionals can navigate the landscape of prone ventilation, optimizing its application and contributing to improving critical care practices.

## References

[1] Hickey SM, Giwa AO (2023). Mechanical ventilation. https://www.ncbi.nlm.nih.gov/books/NBK539742/.

[2] Petrone P, Brathwaite CE, Joseph DK (2021). Prone ventilation as treatment of acute respiratory distress syndrome related to COVID-19. Eur J Trauma Emerg Surg.

[3] Sud S, Friedrich JO, Adhikari NK (2014). Effect of prone positioning during mechanical ventilation on mortality among patients with acute respiratory distress syndrome: a systematic review and meta-analysis. CMAJ.

[4] Guérin C, Albert RK, Beitler J (2020). Prone position in ARDS patients: why, when, how and for whom. Intensive Care Med.

[5] Koulouras V, Papathanakos G, Papathanasiou A, Nakos G (2016). Efficacy of prone position in acute respiratory distress syndrome patients: a pathophysiology-based review. World J Crit Care Med.

[6] Henderson WR, Griesdale DE, Dominelli P, Ronco JJ (2014). Does prone positioning improve oxygenation and reduce mortality in patients with acute respiratory distress syndrome?. Can Respir J.

[7] Zarantonello F, Andreatta G, Sella N, Navalesi P (2020). Prone position and lung ventilation and perfusion matching in acute respiratory failure due to COVID-19. Am J Respir Crit Care Med.

[8] Hepokoski ML, Odish M, Malhotra A (2018). Prone positioning in acute respiratory distress syndrome: why aren't we using it more?. J Thorac Dis.

[9] Cornejo RA, Díaz JC, Tobar EA (2013). Effects of prone positioning on lung protection in patients with acute respiratory distress syndrome. Am J Respir Crit Care Med.

[10] Scholten EL, Beitler JR, Prisk GK, Malhotra A (2017). Treatment of ARDS with prone positioning. Chest.

[11] Morata L, Vollman K, Rechter J, Cox J (2023). Manual prone positioning in adults: reducing the risk of harm through evidence-based practices. Crit Care Nurse.

[12] Douglas IS, Rosenthal CA, Swanson DD (2021). Safety and outcomes of prolonged usual care prone position mechanical ventilation to treat acute coronavirus disease 2019 hypoxemic respiratory failure. Crit Care Med.

[13] Alhazzani W, Parhar KK, Weatherald J (2022). Effect of awake prone positioning on endotracheal intubation in patients with covid-19 and acute respiratory failure: a randomized clinical trial. JAMA.

[14] Ashra F, Chen R, Kang XL (2022). Effectiveness of prone position in acute respiratory distress syndrome and moderating factors of obesity class and treatment durations for COVID-19 patients: a meta-analysis. Intensive Crit Care Nurs.

[15] Bloomfield R, Noble DW, Sudlow A (2015). Prone position for acute respiratory failure in adults. Cochrane Database Syst Rev.

[16] D'Souza FR, Murray JP, Tummala S (2021). Implementation and assessment of a proning protocol for nonintubated patients with COVID-19. J Healthc Qual.

[17] Gohar A, Kirupaharan P, Amaral V, Kokoczka L, Mireles-Cabodevila E, Mucha S, Duggal A (2023). A framework for developing a multidisciplinary approach to prone positioning in acute respiratory distress syndrome. J Intensive Care Med.

[18] Hochberg CH, Card ME, Seth B, Kerlin MP, Hager DN, Eakin MN (2023). Factors influencing the implementation of prone positioning during the COVID-19 pandemic: a qualitative study. Ann Am Thorac Soc.

[19] Anand RK, Baidya DK, Maitra S, Ray BR (2021). A proposal for dedicated “prone team” and “prone bundle of care” in COVID-19 ICU. Indian J Crit Care Med.

[20] Binda F, Galazzi A, Marelli F (2021). Complications of prone positioning in patients with COVID-19: a cross-sectional study. Intensive Crit Care Nurs.

[21] Higgs A, McGrath BA, Goddard C, Rangasami J, Suntharalingam G, Gale R, Cook TM (2018). Guidelines for the management of tracheal intubation in critically ill adults. Br J Anaesth.

[22] Lang EV (2012). A better patient experience through better communication. J Radiol Nurs.

[23] Committee on Family Caregiving for Older Adults; Board on Health Care Services; Health and Medicine Division; National Academies of Sciences, Engineering Engineering, and Medicine (2016). Family caregiving roles and impacts. Families Caring for an Aging America.

[24] Reinhard SC, Given B, Petlick NH, Bemis A (2008). Supporting family caregivers in providing care. https://www.ncbi.nlm.nih.gov/books/NBK2665/.

[25] Wade D, Als N, Bell V (2018). Providing psychological support to people in intensive care: development and feasibility study of a nurse-led intervention to prevent acute stress and long-term morbidity. BMJ Open.

[26] National Clinical Guideline Centre (UK) (2014). Pressure redistributing devices. The Prevention and Management of Pressure Ulcers in Primary and Secondary Care.

[27] Elías MN (2021). Assessment and monitoring of sleep in the intensive care unit. Crit Care Nurs Clin North Am.

[28] Cammarota G, Bruni A, Morettini G (2023). Lung ultrasound to evaluate aeration changes in response to recruitment maneuver and prone positioning in intubated patients with COVID-19 pneumonia: preliminary study. Ultrasound J.

[29] Soriano GP, Yasuhara Y, Ito H (2022). Robots and robotics in nursing. Healthcare (Basel).

[30] Kwee MM, Ho YH, Rozen WM (2015). The prone position during surgery and its complications: a systematic review and evidence-based guidelines. Int Surg.

[31] Harcombe CJ (2004). Nursing patients with ARDS in the prone position. Nurs Stand.

[32] Institute of Medicine (US) Committee on the Health Professions Education Summit (2003). The core competencies needed for health care professionals. Health Professions Education: A Bridge to Quality.

[33] V Babu M, Arumugam MK, Debnath DJ (2021). Simulated patient environment: a training tool for healthcare professionals in COVID-19 Era. Adv Med Educ Pract.

[34] Blackmore A, Kasfiki EV, Purva M (2018). Simulation-based education to improve communication skills: a systematic review and identification of current best practice. BMJ Simul Technol Enhanc Learn.

[35] Mosadeghrad AM (2014). Factors influencing healthcare service quality. Int J Health Policy Manag.

[36] Baston CM, Coe NB, Guerin C, Mancebo J, Halpern S (2019). The cost-effectiveness of interventions to increase utilization of prone positioning for severe acute respiratory distress syndrome. Crit Care Med.

[37] Klaiman T, Silvestri JA, Srinivasan T (2021). Improving prone positioning for severe acute respiratory distress syndrome during the covid-19 pandemic. An implementation-mapping approach. Ann Am Thorac Soc.

[38] Institute of Medicine (2004). Creating and sustaining a culture of safety. Keeping Patients Safe: Transforming the Work Environment of Nurses.

[39] Institute of Medicine (2004). Transformational leadership and evidence-based management. Keeping Patients Safe: Transforming the Work Environment of Nurses.

[40] Guise V, Aase K, Chambers M, Canfield C, Wiig S (2021). Patient and stakeholder involvement in resilient healthcare: an interactive research study protocol. BMJ Open.

[41] Chiu M, Goldberg A, Moses S, Scala P, Fine C, Ryan P (2021). Developing and implementing a dedicated prone positioning team for mechanically ventilated ARDS patients during the COVID-19 crisis. Jt Comm J Qual Patient Saf.

[42] Kaye AD, Okanlawon OJ, Urman RD (2014). Clinical performance feedback and quality improvement opportunities for perioperative physicians. Adv Med Educ Pract.

[43] Zhu L, Ni Z, Zhang Y, Zhan Y, Lan M, Zhao R (2023). Barriers and facilitators of adherence to awake prone positioning: a qualitative study using the COM-B model. BMC Pulm Med.

